# Implementing highly specialized and evidence-based pediatric eating disorder treatment: protocol for a mixed methods evaluation

**DOI:** 10.1186/s13012-015-0231-3

**Published:** 2015-03-28

**Authors:** Jennifer Couturier, Melissa Kimber, James Lock, Melanie Barwick, Gail McVey, Sheri Findlay, Cheryl Webb, Marlene Boettcher, Alison Niccols, Tracy Woodford

**Affiliations:** 1 McMaster University, 1200 Main St. W, Hamilton, Ontario Canada; 2 Stanford University, 401 Quarry Road, Stanford, California USA; 3 Research Institute, The Hospital for Sick Children, 555 University Ave, Toronto, Ontario Canada

**Keywords:** Knowledge translation, Implementation science, Eating disorders, Psychotherapy, Family-based therapy, Fidelity

## Abstract

**Background:**

Eating disorders, which include anorexia nervosa and bulimia nervosa, are common in adolescent females and can have serious emotional and physical consequences, including death. Despite our knowledge about the severity of these illnesses, previous research indicates that adolescent patients are not receiving the best available treatment with fidelity. The main goal of this project is to reduce the knowledge gap between what research indicates is the best known treatment and what is actually delivered in clinical practice. Informed by the National Implementation Research Network model and the Consolidated Framework for Implementation Research meta-theory, our primary study aim is to increase the capacity of Ontario-based therapists to provide family-based treatment, by providing training and ongoing supervision.

**Methods/design:**

We will use a multi-site case study with a mixed method pre/post design to examine several implementation outcomes across four eating disorder treatment programs. We will provide a training workshop on family-based treatment as well as ongoing monthly supervision. In addition, we will assemble implementation teams at each site and coach them by phone on a monthly basis regarding any process issues. Our main outcomes include fidelity to the treatment model using quantitative evaluation of audio-recorded therapy sessions, as well as qualitative analysis of the perceptions of the implementation process using audio-recorded focus groups with all clinicians and administrators involved in the study.

**Discussion:**

To our knowledge, this is the first study to evaluate an implementation strategy for an evidence-based treatment for eating disorders. Challenges to date include obtaining ethics approval at all sites, and recruitment. This research will help to inform future studies on how to best implement evidence-based treatments in this field.

## Background

Eating disorders are common, life threatening, and negatively affect the physical, emotional, and social lives of sufferers and their families. These illnesses, including anorexia nervosa (AN) and bulimia nervosa (BN), are the third most common chronic illness affecting adolescents, after obesity and asthma [1,2]. Due to the complex interplay of physical and psychological symptoms, highly specialized care is needed to treat individuals with an eating disorder. Family-based treatment (FBT) has the strongest evidence base for treating pediatric patients with eating disorders, and this treatment approach is recommended in several guidelines [3-9]. Yet, there is a large gap between this research evidence and the treatments that are actually used in practice [10,11].

Developed at the Maudsley Hospital in London, England, FBT has been manualized by Lock, LeGrange, Agras & Dare [12]. FBT is an outpatient, intensive treatment where the family is the primary resource to re-nourish the affected child. It involves three phases of treatment over 9 to 12 months. The first phase focuses on helping the family to restore the child’s weight and interrupt eating disordered behavior. The second phase involves transition of control of eating behavior back to the adolescent. The third and final phase addresses developmental issues such as physical development, peers and dating, or separation and individuation. This treatment requires that the therapist weigh the patient at each session and suggests that a dietician is not involved, as meal plans are discouraged.

A recent Cochrane review on family therapy for individuals of all ages with AN provides evidence that family therapy may be more effective than treatment as usual [13]. Similarly, a recent meta-analysis [14] has shown FBT to be superior in facilitating full remission of eating disorder symptoms in adolescents at 6- to 12-month follow-up compared to individual treatment, although there were no significant differences in remission rates immediately following treatment. This meta-analysis indicated the same results for AN and BN; that FBT was superior to individual treatment at follow-up. In terms of overall remission rates, Lock and colleagues found that at the end of treatment, 96% of patients no longer met criteria for AN [4]. Moreover, at long-term follow-up, weight gains were maintained [15].

Despite the evidence suggesting that FBT is effective in treating children and adolescents with eating disorders and that FBT has the potential to reduce treatment costs by up to 70% through a reduction in hospitalizations [16], few therapists treating children and adolescents with eating disorders consistently use this therapeutic method, or if they do, it is not practiced with fidelity [10]. The need to test and evaluate contextually appropriate implementation strategies to promote the uptake and implementation of FBT with fidelity is extremely high given the heavy demand for specialized services in pediatric eating disorders. In previous research examining the barriers and facilitators to implementing FBT in practice, 19 of the 40 therapists interviewed reported having received training in the FBT model, with 31 of the 40 therapists (47.5%) reporting that they had read some, or the entire FBT manual. However, none of the therapists practiced the model with fidelity (i.e., including weighing the patient, doing a family meal, and not involving a dietician) [10]. In addition, eating disorder program administrators reported that all therapists in their program would require further training *and* ongoing supervision in order to deliver FBT with fidelity [17].

This paper outlines a study protocol designed to evaluate the implementation of FBT using mixed methodology. Two implementation frameworks are being studied: The National Implementation Research Network (NIRN) model of implementation [18,19] and The Consolidated Framework for Advancing Implementation Research (CFIR) [20]. This project will be the first to test these innovative implementation models within eating disorder treatment services. Two previous studies conducted in Ontario by our group have used the NIRN model to direct change initiatives in child and youth mental health settings [21,22]. We will tailor a combined NIRN and CFIR model to the eating disorder context, taking into account the practitioner and administrator preferences for practice change identified in our previous research [10,17,23]. Additionally, our own research identifies the importance of providing the evidence supporting FBT to those involved in the implementation initiative prior to full movement to implementation, along with the need for consistent support in FBT adoption, skill maintenance, and fidelity [17,23]. Finally, any implementation model needs to be adapted to the current context in order to be successful and sustainable, and therefore, evaluating the over-arching constructs of implementation advocated by the CFIR meta-theory and the NIRN model within the context of eating disorder treatment services will inform us of the components of these previously successful implementation models that are transferable to eating disorder treatment services. Knowing which factors and process innovations are related to successful implementation will have important implications for clinical practice, for eating disorders and other mental health disorders.

### Research questions

1. Does our implementation model lead to FBT implementation success at the participating sites?
2. Which baseline implementation constructs (CFIR) predict implementation success for each therapist?
3. What is the experience of therapists, physicians, and administrators implementing FBT across settings, and how does it inform the implementation model?
4. Which FBT fidelity methods (self, parent, and researcher) demonstrate the highest correlations with fidelity scores generated by the gold-standard approach (i.e., FBT expert)?

## Methods/design

This study received funding from the Canadian Institutes of Health Research. It was reviewed by and has received ethics approval from the Hamilton Health Sciences, Research Ethics Board (HIREB), the St. Joseph’s Care Group Research Ethics Board, the Children’s Hospital of Eastern Ontario Research Ethics Board, and the Health Sciences North, Research Ethics Board. The fourth participating site, Canadian Mental Health Association Waterloo, Wellington, Dufferin, does not have an internal ethics review board.

We will use a multi-site case study with a mixed method pre/post design to examine several implementation outcomes across four child and adolescent eating disorder treatment programs. We will work with four pediatric eating disorder programs affiliated with the Provincial Network for Eating Disorders who provide psychotherapeutic intervention to children and adolescents under the age of 18 years [24]. Four participating organizations were selected from 22 potential organizations within the Ontario Provincial Network for Eating Disorders based on their readiness and motivation to increase their use of and fidelity to the FBT model during our pilot feasibility research. The four partnered treatment programs participating in this study average five therapists per program, yielding an approximate sample size of 20 therapists, four physicians, and four administrators to participate in this study.

### Procedures and timeline

#### Months 0–4: program installation phase: readiness assessment and implementation team formation

Our research approach will follow the implementation stages identified by the NIRN model [19]. Our pilot feasibility research has covered the exploration/adoption phase in which program fit was considered in addition to barriers and facilitating factors for each organization [10], and as such, this project will start at the program installation phase. The formation of the implementation teams during this phase will follow the suggestions by Fixsen et al. [19] and include program staff who are knowledgeable about the treatment being implemented, as well as organizational processes and procedures affecting implementation. For this reason, we will suggest to programs that their implementation teams identify, at a minimum, one therapist, one physician, and one program administrator to participate on the implementation team. In addition, one FBT expert who participated in the delivery of the FBT training program (JC) as well as an implementation expert (MK) will also participate on the implementation team. These suggestions stem from previous studies which have followed the NIRN stages of implementation and have employed the use of implementation teams [21,25].

#### Implementation teams

Members of the research team (JC and MK) will travel to each of the four eating disorder treatment program sites in this phase to formalize implementation team membership; discuss the implementation process and implementation team directives; assess organizational, administrator, and therapist readiness for change; and identify the therapists who will undergo training and supervision in FBT. Each implementation team will meet with the research team three times over this 4-month period; earlier research has demonstrated that implementation teams left on their own, with expert consultation and guidance, are unproductive (Barwick et al., in preparation). At the first meeting, implementation teams will receive guidance on how to communicate information about the implementation project to their respective program staff. At the second meeting, the research team will facilitate, in partnership with the implementation teams, obtaining consent from program staff and collect NIRN process and baseline CFIR construct measures. At the third meeting, engagement of therapists will be discussed.

#### Months 4–5: initial implementation phase: FBT training, training evaluation, and full-implementation preparation

The initial implementation phase of the NIRN model is the stage at which changes begin to occur to the overall practice environment, including the skill level of therapists and overall program capacity. During this time, all therapists, administrators, and physicians will undergo training in the FBT intervention model.

#### Implementation teams

The implementation teams will continue to meet the research team on a monthly basis; however, these meetings will now be conducted over the phone (implementation coaching calls). In addition, the implementation teams will be encouraged to meet on a monthly basis away from the research team to address issues discussed on the coaching calls, sustainability and fidelity to the FBT model, and will be asked to report back to the research team at the monthly coaching calls about the number of formal meetings which have taken place since the last implementation team coaching call. As per recent NIRN guidelines, implementation teams will be asked on each coaching call for the remainder of the study to share what they have considered as important factors within their program to sustain FBT use within their program over time and what innovations to the FBT model may be necessary for sustained FBT use among their therapists.

#### FBT training and supervision

FBT training will be conducted at a central location and will consist of 2 days of training led by JL, and involving JC, SF, and CW. Participants will receive an orientation to the overall project and complete a demographic information sheet. The duration and content of training will be informed by the training model employed by *The Training Institute for Child and Adolescent Eating Disorders* in Chicago, IL, and the expertise of JL (first author of the FBT treatment manual and co-director of the training institute) and will include a package containing all research articles related to the effectiveness of FBT and the FBT treatment manual. Implementation team members will also be encouraged to attend the training. Therapists will then receive an FBT implementation toolkit, which will describe the implementation process, including clinician responsibilities for participation in the implementation endeavor, as well as all of the necessary materials for the submission of FBT fidelity assessments and feedback, including audiotapes and a recorder. Baseline CFIR construct measures will be collected prior to the training workshop, for those who are not members of implementation teams.

#### Months 6–18: full operation: implementation of FBT in practice

The full operation phase of the NIRN model is the point at which the new treatment becomes integrated into practitioner and program practices. Additionally, fidelity can be evaluated at this stage.

#### Implementation teams

Implementation team members will continue to undergo monthly teleconferences with the research team to discuss ongoing implementation issues. Implementation teams will also continue to meet monthly without the research team.

#### FBT training/coaching and supervision

Throughout the *full operation* phase, therapists will provide a monthly audio sample of an FBT session to the research team (reviewed by JC and MK), will receive FBT fidelity feedback from the research team, and will participate in monthly clinical FBT group coaching/supervision calls by an FBT trainer (JC) to troubleshoot general clinical FBT issues. Therapists will be encouraged to meet independently from the research team for peer supervision on a monthly basis.

#### Months 18–24: evaluation: assessing the experience of the implementation intervention

The evaluation phase of our implementation intervention involves the solicitation of feedback about the overall implementation process and a consideration of potential innovations to the FBT model that may be perceived as necessary for sustained use over time.

#### Implementation teams

Implementation teams will continue to meet on a monthly basis without the research team.

#### FBT training and supervision

Therapists will continue to meet on a monthly basis for peer supervision without the research team. The research team will have no contact with the participating organizations from months 18 to 21 so that the participating programs are left to sustain FBT implementation on their own for a period of 3 months. From month 21 to 24, eight focus groups will be completed in this stage of the study. Two focus groups will be completed at each organization, with one of these involving the therapists implementing the FBT model, and the other including implementation team members. Focus group questions will elicit experiences of the implementation project, perceptions about implementation success, and needed areas for innovation.

### Measures

#### Question #1

Organizational level FBT implementation success will be defined as 80% of therapists at each site rated as demonstrating 80% fidelity to the model at each session. Fidelity of each audio-recorded therapy session will be rated by an FBT expert (JC) using the FBT fidelity measure developed by James Lock and piloted in previous studies [26,27]. Each therapist will record one patient and submit sessions #1, 2, and 3, from phase 1, along with monthly sessions thereafter.

#### Question #2

Individual therapist implementation success will be determined by achieving fidelity ratings of 80% or greater on each assessed session. We will focus on evaluating ten of the 31 CFIR constructs in light of recent research by Damschroder & Lowery [28] that has identified ten constructs as the most strongly associated with implementation effectiveness. These ten constructs, as well as other implementation-related factors will be quantitatively assessed by administering various measures at baseline to all therapists, administrators, and physicians participating in the study. The construct of organizational readiness will be assessed using a modified version of the short form of the Organizational Readiness for Change Scale (ORC). A recent review indicates that the ORC is the most widely used measure of organizational readiness and, for our purposes, also assesses the CFIR constructs of 1. tension for change, 2. networks and communication, 3. relative priority, and 4. organizational culture [29]. The CFIR construct of clinician readiness for change will be assessed using the Brief Individual Readiness for Change Scale (BIRCS) [30]. Clinician and administrator attitudes about Evidence-Based Practice will be assessed using the Evidence-Based Practice Attitudes Scale (EBPAS) [31,32]. The EBPAS assesses mental health provider attitudes toward adoption and innovation in mental health services. The CFIR construct of learning climate will be evaluated by administering to staff the Organizational Learning Survey [33]. Scores on this measure will be used to inform our understanding about implementation success and fidelity at follow-up time points. And finally, CFIR constructs of 1. relative advantage, 2. trialability, and 3. complexity will be assessed by administering an adapted version of the *Perceived Attributes of the Principles of Effectiveness* [34] measure; whose language has been edited to elicit therapists perceptions of the FBT model fit with their program.

#### Question #3

The experience of therapists, physicians, and administrators implementing FBT across settings will be evaluated qualitatively. These qualitative evaluations will focus on three CFIR constructs which include perceptions of 1) the execution of the implementation, 2) the overall success of the implementation, and 3) receipt of feedback throughout the implementation process. Therapists, physicians, and administrators will participate in a final evaluation focus group, which will be audio recorded, transcribed verbatim, and analyzed. Implementation processes will also be assessed by audio-recording implementation team meetings and coaching calls, and transcribing these recordings verbatim to inform our evaluation of the overall implementation intervention. In addition, we will record and transcribe all clinical supervision calls.

#### Question #4

The literature suggests that the level of a clinician’s knowledge about a treatment should correlate with their level of adherence (i.e., fidelity); and for this reason, a fidelity measure can be both a measure of competence as well as fidelity [35]. The FBT fidelity measure will be completed by each therapist (self report) for each session that they are submitting for review. The therapist will also ask the parent to fill out the measure corresponding to the session. Sessions will then be rated by a researcher (MK) and a clinical FBT expert (JC) for fidelity.

### Analysis

#### Question #1

The percentage of therapists demonstrating FBT fidelity will be determined by using the FBT fidelity rating scale and achieving a 4/5 average score or greater on each session.

#### Question #2

Factors predicting implementation success will be assessed using logistic regression, with implementation success for each therapist as the dependent variable (achieving 80% average fidelity ratings on each session), and baseline CFIR constructs as independent variables. We will also examine demographic characteristics including sex, gender, and age in relation to implementation success.

#### Question #3

Transcripts resulting from the implementation team meetings and coaching calls, clinical FBT training workshop and supervision calls, and the innovation and evaluation focus groups will be analyzed using interpretive description [36]. The process of interpretive description is uniquely suited to multiple sources of data by its ability to allow researchers to make links between the particular and the general within and across data sources [37-39]. Therefore, we will follow the approach of Damschroder & Lowery [28] in using both inductive and deductive interpretive description analysis techniques with our qualitative data; the intention of which is to identify any emergent and unique (inductive analysis) process themes that are specific to FBT implementation in pediatric eating disorders, but also, to evaluate the extent to which the implementation experience and processes in our project map onto what has been described in other studies employing the CFIR and NIRN frameworks (deductive analysis).

#### Question #4

A number of analyses will be undertaken in an effort to validate the FBT fidelity measure. We will complete an inter-rater reliability analysis examining the correlations between therapist-reported, parent-reported, researcher-reported, and FBT expert-reported fidelity scores as well as examine the agreement among subscale ratings on the measure.

### Trial status

At the time of manuscript submission, the study is in the full operation phase of the NIRN model where FBT is being integrated into practitioner and program practices. No data cleaning or analysis has been performed.

## Discussion

The purpose of this study as outlined in this protocol is to evaluate the processes involved in the uptake and use of FBT to treat children and adolescents with eating disorders using an implementation framework novel to the field of pediatric eating disorders. This is the first project to evaluate a combined NIRN model and CFIR meta-theory specifically within eating disorder treatment services, thereby contributing to our knowledge base about the external validity of these frameworks across different fields. The results of the study will form the basis for the future implementation of FBT across Ontario, providing insight into the efficacy of this innovative framework to increase the uptake of FBT within eating disorder programs, as well as to further our understanding of the fundamental mechanisms requisite for successful implementation of this treatment. Ongoing support is thought to be necessary for the successful implementation of any psychotherapeutic approach. Ultimately, our goal is to increase access to FBT for all Canadian children and adolescents with eating disorders, thereby improving recovery rates and reducing the need for hospitalization.

At the time of submission of this protocol, we are in the full operation phase of the study. Certain challenges have been met thus far and are worthy of discussion. In terms of ethics approval, our institution provided initial approval, and then ethics procedures for all of the four sites had to be examined and followed. This was a lengthy process, taking several months. Approval from the other sites then had to be submitted back to our own board at McMaster University. Each of the boards had their own unique concerns in terms of sessions being recorded for the fidelity ratings, as well as unique requirements for letters of consent.

An additional challenge has been the distance to the sites, with three of the four sites requiring a flight to make a site visit. The furthest site, Thunder Bay, Ontario, is 925 km away. We had initially planned on making three visits to each site prior to implementing the training event; however, it was difficult to schedule a meeting that was convenient to the implementation team at each of these sites and the researchers. We were forced to change the protocol and only made one site visit to each of the four sites.

Recruitment for the study has been multi-layered. During the development of the grant proposal four sites had agreed to participate. When final approval and funding came through, one of the sites declined to participate. This was a large academic center and was replaced with a smaller community clinic. This was probably a better choice, as the smaller clinic is more representative of community-based practice and our results will be more generalizable to those settings. After the sites were finalized, the implementation team including the administrator, medical role, and lead therapist had been recruited. Finally, other therapists had to be recruited at each site by the lead therapist. Following this, families were approached by the therapists at each site and were required to sign consent for the audio recordings and the fidelity measures. This multi-staged approach has been time-consuming and relies heavily on the sites, which is outside the control of the researchers. Despite these challenges, all sites have recruited families and the implementation and supervision calls are ongoing on a monthly basis.

In summary, this is the first study to evaluate an implementation strategy involving FBT in pediatric eating disorders. Challenges to date include obtaining ethics approval for this multi-site study, great distances that must be traveled to our sites, and our multi-layered recruitment. Results from this study will provide valuable information of the usefulness of the NIRN and CFIR in providing a common implementation framework that allows comparison of key constructs across different studies and contexts; something that is greatly needed if we are to move the field forward. In addition, this research will help to inform further studies on how to best implement evidence-based treatments in the field of eating disorders. Future studies should include an economic component to assess the cost effectiveness of this model of implementation.

## Abbreviations

BIRCS: Brief Individual Readiness for Change Scale
CFIR: Consolidated Framework for Advancing Implementation Research
EBPAS: Evidence-Based Practice Attitudes Scale
FBT: Family-based treatment
NIRN: National Implementation Research Network
ORC: Organizational Readiness for Change Scale
